# Association of WHSC1/NSD2 and T-cell infiltration with prostate cancer metastasis and prognosis

**DOI:** 10.1038/s41598-023-48906-8

**Published:** 2023-12-07

**Authors:** Qiheng Li, Jiang Zhu, Yang Zhang, Yun Pan, Zhengjin Li, Min Wang, Yixuan Gao, Dongmei Feng, Xiaoyong He, Chunmei Zhang

**Affiliations:** 1 Department of Pathology, The First Affiliated Hospital of Dali University, Yunnan, China; 2 Department of Urology Surgery, The First Affiliated Hospital of Dali University, Yunnan, China; 3 Department of General Surgery, The First Affiliated Hospital of Dali University, Yunnan, China; 4 Department of Oral and Maxillofacial Surgery, The First Affiliated Hospital of Dali University, Yunnan, China

**Keywords:** Cancer microenvironment, Urological cancer

## Abstract

Progress in immunotherapy for prostate cancer (PCa) lags that for other cancers, mainly because of limited immune infiltration in PCa. This study aimed to assess the feasibility of NSD2 as an immunotherapeutic target in PCa. Immunohistochemistry was performed to evaluate the expression pattern of NSD2 in 34 cases of benign prostatic hyperplasia (BPH), 36 cases of prostatic intraepithelial neoplasia (PIN), and 57 cases of PCa, including 19 cases of metastatic castration-resistant prostatic cancer (mCRPC). Single-cell RNA sequencing and gene set enrichment analysis (GSEA) were used to correlate NSD2 with certain downstream pathways. Furthermore, the Immuno-Oncology-Biological-Research (IOBR) software package was used to analyze the potential roles of NSD2 in the tumor microenvironment. We found that the positive expression rate of NSD2 increased progressively in BPH, PIN and PCa. mCRPC had the highest staining intensity for NSD2. High NSD2 expression was positively correlated with the infiltration level of CD4^+^ tumor-infiltrating lymphocytes (TILs) and negatively correlated with that of CD8^+^ TILs. Importantly, a new immune classification based on NSD2 expression and CD4^+^ TILs and CD8^+^ TILs was successfully used to stratify PCa patients based on OS.PSA and CD4^+^ TILs are independent risk factors for PCa bone metastasis. This study demonstrates a novel role for NSD2 in defining immune infiltrate on in PCa and highlights the great potential for its application in immunotherapy response evaluation for prostate malignancies.

## Introduction

Prostate cancer (PCa) is the most commonly diagnosed cancer in men and is one of the leading causes of cancer-related death worldwide^1^. PCa growth is intrinsically driven by the androgen receptor (AR), and androgen deprivation therapy (ADT), which blocks androgen signaling, is a common therapeutic intervention aimed at reducing tumor size^2^. ADT induces the formation of a complex proinflammatory tumor microenvironment. This pro-inflammatory microenvironment is apparent in the early post castration period, but the majority of PCa cases ultimately progress to castration-resistant prostatic cancer^3^. Castration-resistant prostate cancer (CRPC) is incurable and has a poor prognosis as it often metastasizes to bones, lymph nodes or bladders. This advanced stage is called mCRPC. However, once recurrence and distant metastasis occur, which means that the patient is resistant to current treatment, the disease progresses rapidly, eventually leading to death^4^. For localized PCa, radical prostatectomy with/without radiation is the main therapeutic strategy, but treatment options for CRPC have been limited for decades^5^. In light of this, there is a pressing need to assess new and existing immunotherapy regimens to increase treatment efficacy.

With the intensive investigation of the immune microenvironment within tumors, it is evident that immune check‐point inhibitors (ICIs), which counteract the functional suppression of CD8^+^ cytotoxic T cells (CTLs), have produced some unprecedented long‐lasting responses in cancer immunotherapy. ICI treatment is especially effective in “hot” tumors containing high numbers of CTLs^6^. However, the efficacy of ICIs in treating PCa has been disappointing^7^. A better understanding of PCa TILs and their microenvironment may improve our ability to target PCa immunologically. Many reports provide evidence of the favorable prognostic implications value of TILs in many cancers, but the role of TILs in PCa and their composition remain controversial^8,9^.

NSD2 is a histone methyltransferase, elevated NSD2 expression correlates with worse prognosis in a number of cancers due to its oncogenic role in promoting cell growth and metastases. However, the magnitude of the effect and mechanism(s) of action of NSD2 remain poorly understood^10,11^. NSD2 is associated with diseases affecting growth and development and plays a role in the DNA damage response^12,13^. A study suggests a novel role for NSD2 in the immune infiltration of prostate cancer, with elevated levels of the WHSC1 enzyme limiting lymphocyte infiltration in prostate cancer tumours, and positively correlating with the presence of an immunosuppressive microenvironment^14^. Subsequent studies found that NSD2 inhibition increased phenotypic transformation of immune cells, such as infiltration of cytotoxic T cells and NK cells and local regulation of different myeloid cell subpopulations present within the tumour, as well as translocation of key signalling pathways^15^. Studies in cervical cancer revealed that NSD2 promotes tumor progression and metastases by inducing transforming growth factor-beta (TGF-β)-mediated immunosuppression^16^. Consistently, the upregulation of NSD2 is correlated with the expression of IL-6 and TNF-α in the prostate^17^. Moreover, in colorectal cancer, NSD2 loss also affects the release of IFN-γ through MHC-I, thus suppressing the immune response^18^. These apparent discrepancies may potentially be associated with the composition of tumor-infiltrating immune cells (TIICs) within different tumors.

Here, we compare the immunohistochemical profiles in a series of 57 cases of PCa, 34 cases of benign prostatic hyperplasia (BPH), 36 cases of prostatic intraepithelial neoplasia (PIN), and 19 cases of mCRPC in order to obtain a more complete understanding of the immunostaining profile of NSD2 in prostate cancer and to further examine its potential as a biomarker for PCa and/or mCRPC.

The aim of this retrospective study was to evaluate the expression pattern and clinical significance of NSD2 and its correlations with TILs in PCa.

## Methods

### Patients and specimens

We conducted a retrospective study of patients providing paraffin-embedded surgical tissue specimens; the specimens were from 34 cases of BPH, 36 cases of PIN and 57 PCa cases and were collected in the Department of Pathology at First Affiliated Hospital of Dali University (Dali, China). A total of 19 patients presented with distal metastasis specimens, and to be eligible, the patients were required to have metastatic disease detected on conventional imaging studies, including computed tomography (CT) scans, magnetic resonance imaging (MRI) or bone scans. Patients were also required to have castration-resistant disease.

Data from eligible patients were retrospectively abstracted from the electronic medical records following acquisition of institutional review board approval. Data of interest included age, history of drinking, any prior local therapy to the prostate, Gleason score, ISUP grade, sites of metastasis and pTNM stage. In addition, we referred to the baseline laboratory records to collect data on blood counts, results of any BM aspirate and biopsy, serum PSA, and neutrophil to lymphocyte ratio (NLR). Finally, we gathered dates of last follow-up or death. Overall survival (OS) was calculated from prostatectomy to death or the last follow-up, which was August 1, 2022.

Formalin-fixed, paraffin-embedded tissue blocks were available from all cases, and H&E-stained tumor slides were used to confirm the Gleason score, tumor stage and lymph node status by two senior pathologists according to the 2005 International Society of Urological Pathology (ISUP) Consensus conference^19^. Imaging examination was used to determine distal metastasis. Tumor stage and grading were evaluated according to the 2002 American Joint Committee on Cancer (AJCC) TNM classification. Patients were followed up every 3 months for serum PSA assessment and, if possible, imaging studies (computed tomography, bone scan, or magnetic resonance imaging).

This study was approved by the Ethics Committee of the First Affiliated Hospital of Dali University. All patients provided written informed consent, and the study was conducted in accordance with the Declaration of Helsinki.

### Immunohistochemistry

Formalin-fixed PCa tissue sections were deparaffinized in xylene and hydrated in a diluted alcohol series. Then, antigen retrieval was accomplished with EDTA buffer (0.5 mM, pH 8.0) using a microwave for 20 min. Following blocking with goat serum, the sections were incubated with corresponding primary antibodies (anti-NSD2: Abcam, 1:400, #ab75359; anti-CD4: Maixin, RMA-0620, ready‑to‑use; anti-CD8: Maixin, MAB-0021, ready‑to‑use) at 4 °C overnight. Then, after the sections were washed three times using phosphate-buffered saline (PBS). Finally, the sections were then incubated with EnVision Detection Systems Peroxidase/DAB, Rabbit/Mouse (Dako; Agilent Technologies, #K5007; ready‑to‑use) secondary antibodies for 30 min at room temperature. DAB from the aforementioned secondary staining kit was added for detection. Sections were counterstained with hematoxylin for 2 min at room temperature.

### Assessment of NSD2, CD4^+^ and CD8^+^ TILs expression and the infiltration level of infiltrated immune cells

All sections were assessed by two independent pathologists blinded to the clinical profiles of the patients, and discrepancies were resolved after consensus. The Human Protein Atlas database was further used to analyze the differential protein expression of NSD2 in PCa and normal prostate tissues. The expression was defined using the criteria set in the database. The NSD2 protein expression score is based on immunohistochemical data manually scored with regard to staining intensity and fraction of stained cells. In detail, the staining intensity was divided into four levels as follows: absent (0), weak (1), moderate (2) and strong (3). The fraction of stained cells was divided into four levels as follows: < 10 (1), 11–50 (2), 51–80 (3) or ≥ 80% (4). The H-score of each case was determined by multiplying the staining intensity by the corresponding percentage of positive cells. The H-score ranged from 0 to 12. An H-score > 0 was defined as positive expression, and the cutoff value for differentiating high and low NSD2 expression was 6.

The infiltration extent of CD4^+^ and CD8+ TILs infiltration was evaluated according to the mean counts in ten independent high-power fields (400×) within the mesenchyme, which represented the densest immune cell infiltrates. The mean value was considered the cutoff point for group stratification in this study^20^.

### Bioinformatics analysis

We downloaded PCa data from the GSE153333 dataset of the GEO database (https://www.ncbi.nlm.nih.gov/geo/query/acc.cgi?acc=GSE153333), which included information on NSD2 knockout (NSD2-KO) and CTR C42 cells^14^, and performed differential analysis using the DESeq package to obtain 1867 differential genes with |logFC| > 0.5.

Gene set enrichment analysis (GSEA)^21,22^ of differentially expressed genes in the GEO dataset was performed using the package clusterProfiler^23^. Canonical pathways significantly altered by NSD2 knockout were determined by GSEA (http://software.broadinstitute.org/gsea/index.jsp) based on the hallmark gene set and KEGG gene sets from the Molecular Signatures Database (MSigDB) (https://www.gsea-msigdb.org/gsea/msigdb).

The Immuno-Oncology-Biological-Research (IOBR) package in R integrates 5 commonly used algorithms (MCPcounter, xCell, CIBERSORT, EPIC and quanTiseq) to separately analyze TILs in the TME based on 255 gene signatures related to tumors and the TME^24^. The ESTIMATE algorithm was used to determine the immune score and ESTIMATE score for tumors. xCell conducts cell type enrichment analysis using gene expression data for 64 immune and stromal cell types. To minimize the correlation between closely linked cell types, xCell uses machine learning based on gene signatures from thousands of different cell types. By validating extensive computer simulations of signature subtyping and cellular immunophenotyping, xCell is able to reliably map the cellular heterogeneity of the tissue based on the expression profile.

### Statistical analysis

The distribution of clinicopathological parameters, including the densities of different sets of immune cells over NSD2 expression categories, was evaluated using χ^2^ or Fisher’s exact test. The Spearman rank correlation test was used to analyze the expression correlation between NSD2, CD4^+^ and CD8^+^ TILs. The OS curves were depicted with the Kaplan‒Meier method. Logistic regression analysis was used to analyze the related risk factors for prostate cancer bone metastasis, and Cox proportional hazard regression analysis was performed for univariate and multivariate analyses. Differences were considered statistically significant at P < 0.05. All statistical analyses were performed using SPSS V.26.0 (Chicago, Illinois, USA) or GraphPad Prism V.9 (La Jolla, California, USA) software.

### Institutional review board statement

The study was conducted according to the guidelines of the Declaration of Helsinki, and approved by the Ethics Committee of the First Affiliated Hospital of Dali University (approval number: 20220412).

### Informed consent

Informed consent was obtained from all subjects involved in the study.

## Results

### Clinicopathological characteristics of patients with BPH, PIN and PCa

**Figure 1:**
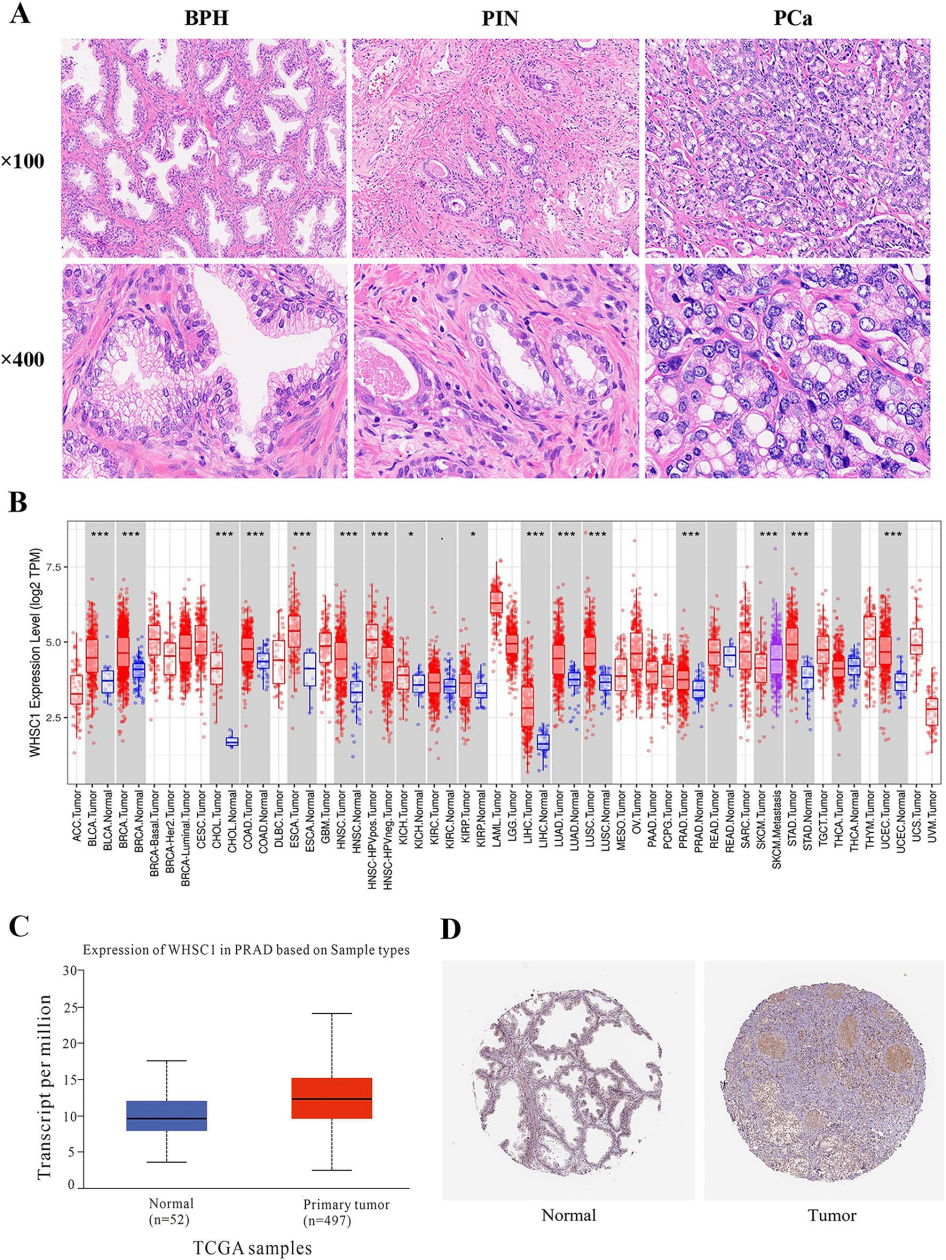
NSD2 proteins were overexpressed in PCa tissues. (**A**) HE staining of tissue sections: BPH (×400), PIN (×400) and PCa (×400). (**B**) The expression status of the NSD2 gene in different cancers or specific cancer subtypes was analyzed through TIMER2. (**C**) The expression levels of NSD2 between normal and PCa tissues were analyzed by UALCAN. (**D**) NSD2 expression in normal prostate tissue and prostate cancer was analyzed using the Human Protein Atlas database.

First, 34 cases of BPH, 36 cases of PIN and 57 cases of PCa were screened(Fig. 1A). The basic clinicopathological characteristics of 57 patients with PCa are summarized in Table 1. The mean age at operation was 71 years (range, 53–87), of whom 52 patients (91.2%) were aged > 60 years and 5 patients (8.8%) were aged ≤ 60 years. Among the patients, 14 patients (25%) had a history of alcohol consumption . 41 patients (71.9%) were in the Gleason score range of 6–7(3 + 4) and 16 patients (28.1%) were in the range of 7(4 + 3)−10 interval in 16 patients (28.1%).Serum PSA levels were predominantly > 20, accounting for 63.2%. The levels of preoperative and postoperative NLR were predominantly low-grade, accounting for 80.7% and 60.0%, respectively. TNM staging: ≤ T2 stage 30 cases (52.6%), T3-4 stage 27 cases (47.4%), N0 stage 42 cases (73.7%), N1 stage 15 cases (26.3%), M0 stage 38 cases (66.7%), M1 stage 19 cases (33.3%). At the end of follow-up, 14 (24.6%) patients had died, and of these patients, 10 (52.6%) patients presented distant metastasis. The median follow-up time was 14 months (range, 5–42). The OS rate was 75.4%.

**Table 1:** Basic characteristics of prostate cancer patients.

Variables	Patient numbers n (%), total n = 57
Age(year)	
≤ 60	5 (8.8%)
> 60	52 (91.2%)
History of drinking	
Yes	14 (25%)
No	42 (75%)
Gleason grade	
6–7 (3 + 4)	41 (71.9%)
7 (4 + 3)–10	16 (28.1%)
ISUP	
1	9 (15.8%)
2	7 (12.3%)
3	13 (22.8%)
4	11 (19.3%)
5	17 (29.8%)
PSA(ng/ml)	
≤ 20	21 (36.8%)
> 20	36 (63.2%)
Preop NLR	
Low	46 (80.7%)
High	11 (19.3%)
Postop NLR	
Low	21 (60.0%)
High	14 (40.0%)
Tumor stage	
≤ T2	30 (52.6%)
T3–4	27 (47.4%)
Nodal metastasis	
Absent	42 (73.7%)
Present	15 (26.3%)
Metastasis	
Yes	19 (33.3%)
No	38 (66.7%)
Survival statue	
Dead	14 (24.6%)
Alive	43 (75.4%)

### NSD2 expression in BPH, PIN and PCa

**Figure 2:**
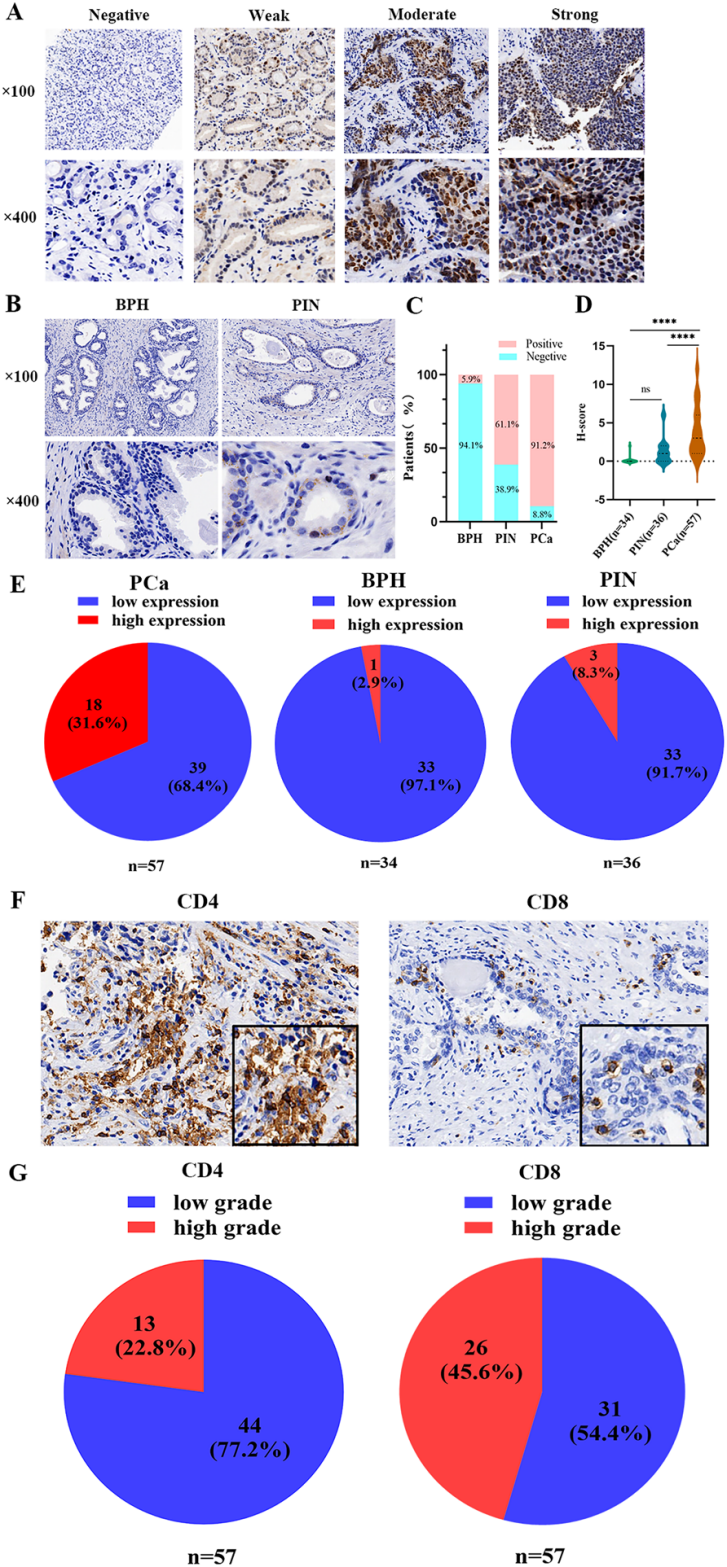
NSD2, CD4^+^ and CD8^+^TILs marker expression in PCa tissue samples. (**A**) Representative immunohistochemical staining of NSD2 within tumors. (**B**) Representative immunohistochemical staining of NSD2 within BPH and PIN. (**C**) NSD2 expression rate in BPH, PIN and PCa. (**D**) Relationship of NSD2 expression in BPH,PIN and PCa. (**E**) NSD2 expression percentage in BPH, PIN and PCa. (**F**) Representative immunohistochemical staining of CD4^+^ and CD8^+^ TILs within tumors. (**G**) The percentage of CD4^+^ and CD8^+^ TILs marker expression in PCa tissue samples.Scale bar, 50 μm.

NSD2 expression between tumor and normal tissues from patients with different cancers was analyzed using the TIMER2.0 database (Fig. 1B). NSD2 overexpression was observed in prostate cancer tissues compared with corresponding normal tissue samples. The expression of NSD2 in PCa was also explored in the TCGA analysis module of the UALCAN database. NSD2 was found to be significantly upregulated in PCa tissues compared with normal tissues (Fig. 1C). In addition, analysis using the Human Protein Atlas database revealed that NSD2 was predominantly located in the cell nucleus (Fig. 1D). Immunohistochemistry was performed to investigate the protein expression patterns of NSD2 in PCa, BPH and PIN tissues, and NSD2 showed a nuclear staining pattern(Fig. 2A,B). NSD2 protein was expressed in the nucleus, with 91.2% (52/57) positive expression of NSD2 in 57 PCa, 5.9% (2/34) positive expression of NSD2 in 34 BPH and 61.1% (22/36) in 36 PIN (Fig. 2C). Immunoprotein scores for NSD2 in PCa were significantly higher than those in BPH and PIN, and there was a significant difference between both BPH and PCa, PIN and PCa (p < 0.0001) (Fig. 2D). The difference in NSD2 expression in PIN and PCa was significant (p < 0.05) (Table 2). Moreover, significant differences were found between BPH and PIN and between BPH and PCa (p < 0.05 in all cases) (Table 2). NSD2 was highly expressed in 18/57 (31.6%) PCa, 1/34(2.9%)BPH and 3/36(8.3%)PIN cases, respectively (Fig. 2E). These data indicate that NSD2 is expressed more frequently in PCa than in BPH and PIN.

**Table 2:** NSD2 protein expression in BPH, PIN and PCa.

Tissue sample	n	NSD2 expression		P-value
		Positive (%)	Negative (%)	
BPH	34	2 (5.9)	32 (94.1)	< 0.0001***
PIN	36	22 (61.1)	14 (38.9)	
BPH	34	2 (5.9)	32 (94.1)	< 0.0001***
PCa	57	51 (89.5)	6 (10.5)	
PIN	36	22 (61.1)	14 (38.9)	0.0012**
PCa	57	51 (89.5)	6 (10.5)	

Significant values are in bold.

### NSD2 expression is correlated with PCa immune infiltration levels

To investigate the protein expression patterns of NSD2 and CD4^+^ and CD8^+^ TILs markers in PCa tissues, immunohistochemistry (IHC) was performed, and CD4^+^ and CD8^+^ TILs showed a cell membrane cytoplasm attaining pattern (Fig. 2F). NSD2 was widely expressed in 52/57 (91.2%) PCa cases. Moreover, CD4^+^ and CD8^+^ TILs markers were expressed in all 57 (100%) PCa cases. The proportions of PCa samples with high CD4^+^ and CD8^+^ TILs infiltration were 13/57 (22.8%) and 26/57 (45.6%), respectively (Fig. 2G).

**Figure 3:**
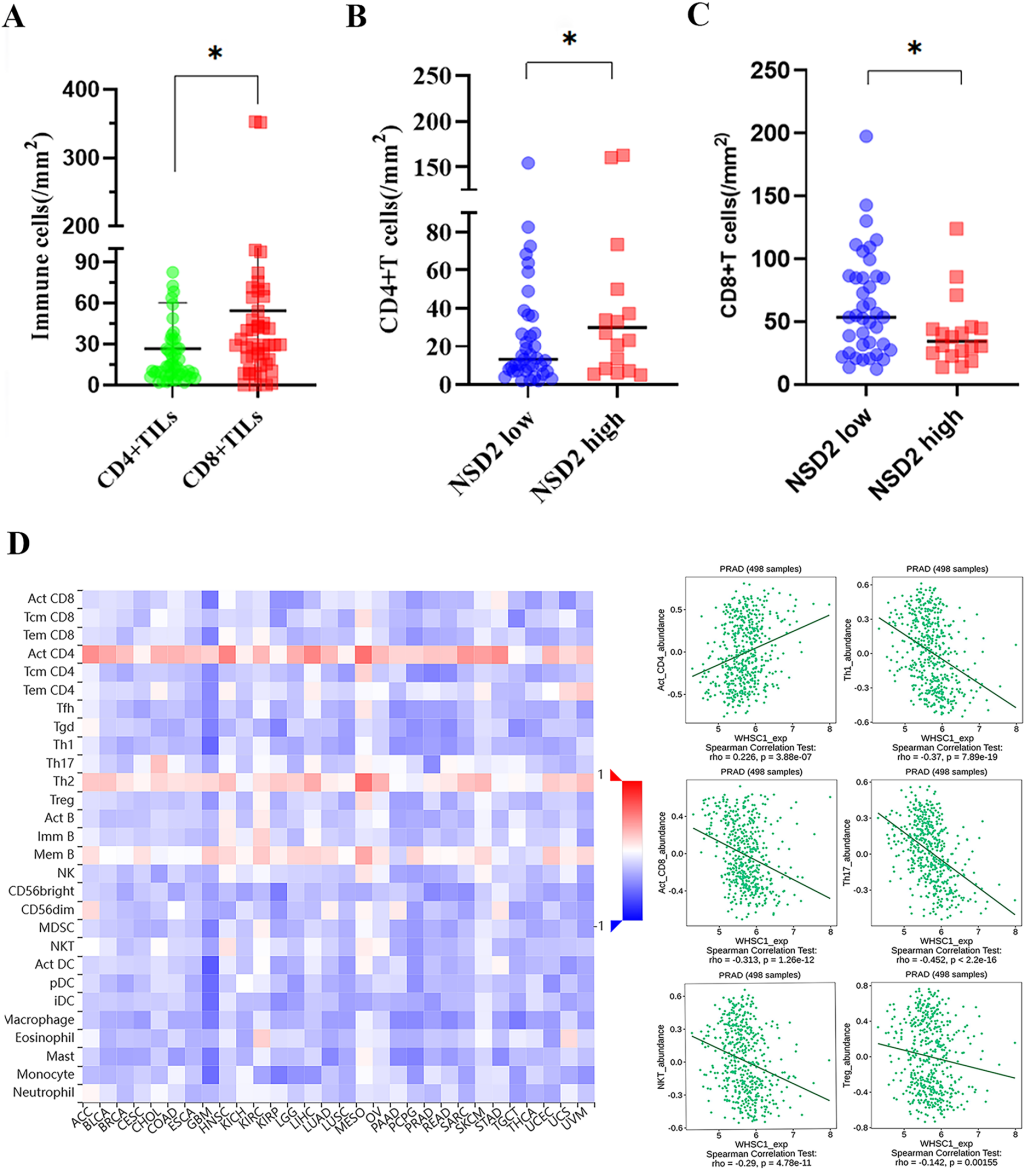
The association of NSD2 expression with the levels of TILs. (**A**) Scatter plot depicting the infiltration level of CD4^+^ or CD8^+^TILs. (**B**) The counts of CD4^+^ TILs in tumors with different NSD2 expression levels. (**C**) The counts of CD8^+^ TILs in tumors with different NSD2 expression levels. (**D**) The relationship between NSD2 expression and lymphocytes was analyzed by TISIBD.

The density of CD8^+^ TILs was much higher than the infiltration level of CD4^+^ TILs in PCa (p = 0.0138, Fig. 3A). The CD4^+^ TIL infiltration level was significantly lower in tumors with low NSD2 expression than in tumors with high NSD2 expression (NSD2, p = 0.0395; Fig. 3B). Conversely, the infiltration level of CD8^+^ TILs was higher in tumors with low NSD2 expression than in tumors with high NSD2 expression (NSD2, p = 0.0479; Fig. 3C). These results suggest that NSD2 is involved in the regulation of the immune microenvironment within PCa tissues.

We explored the relationships between the NSD2 expression level and immune components, such as lymphocytes, in patients with PCa using the TISIDB tool to obtain a deeper understanding of the association of NSD2 with immune cell infiltration. The relationships between the abundance of TILs and NSD2 expression levels were investigated to examine which types of TILs might be regulated by the NSD2 gene. The results indicated that the NSD2 expression level was positively correlated with the infiltration of Act_CD4 cells (rho = 0.226, P = 3.88e−07), Act_CD8 cells (rho = − 0.313, P = 1.26e−12), NKT cells (rho = − 0.29, P = 4.78e−11), Th1 cells (rho = − 0.37, P = 7.89e−19), Th17 cells (rho = − 0.452, P < 2.2e−16) and Treg cells (rho = 0.142, P = 0.00155)(Fig. 3D). The results are strongly consistent with our findings suggesting that NSD2 regulates various immune molecules in the tumor microenvironment of PCa, thereby affecting CD4^+^ and CD8^+^ T immune cell infiltration.

### Association between NSD2, CD4^+^ and CD8^+^ TILs and clinicopathological factors in patients with PCa

**Figure 4:**
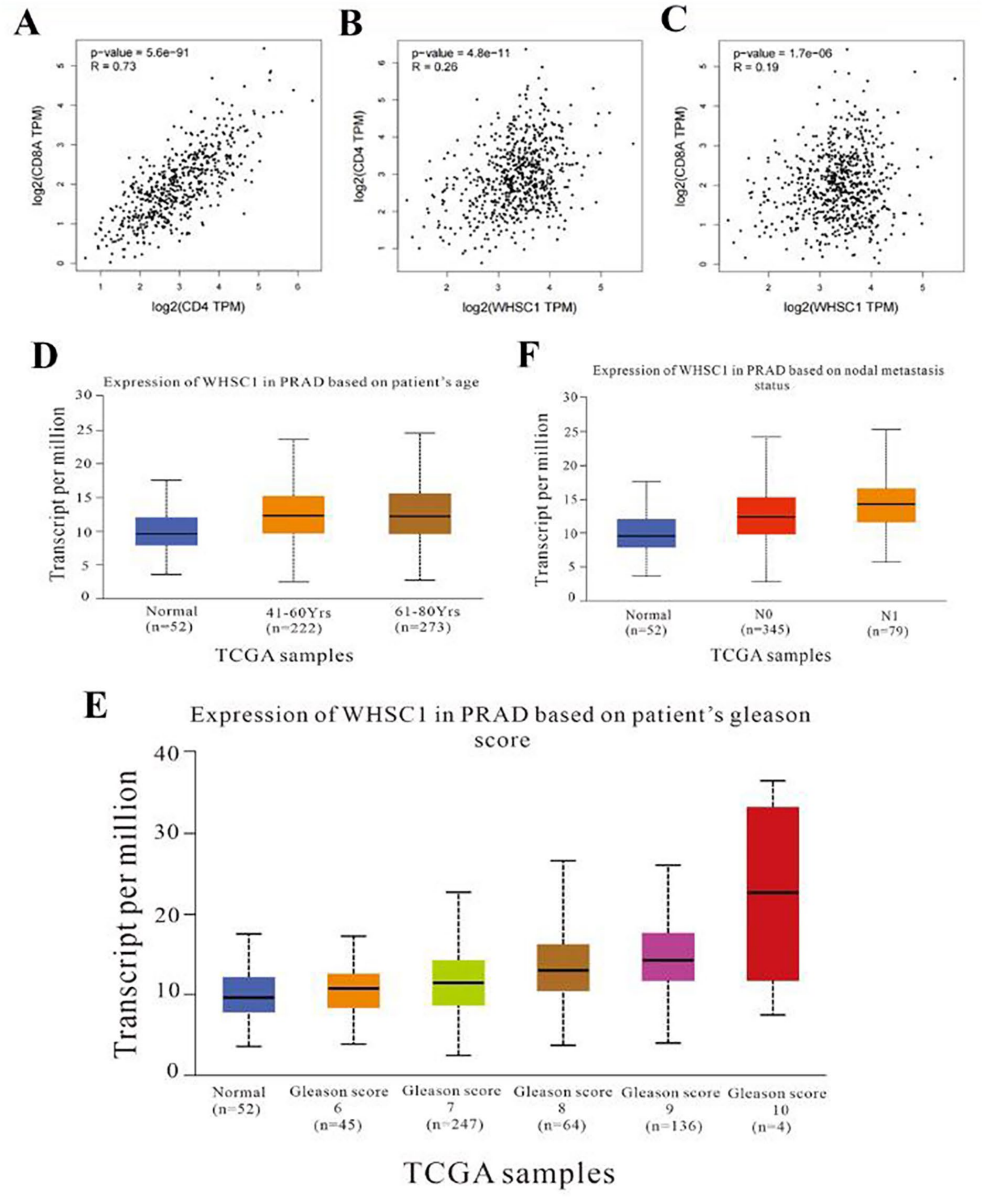
Expression level of the NSD2 gene in groups based on clinicopathological features. (**A**–**C**) The correlation of NSD2, CD4 and CD8A gene expression in PCa was analysed by GEPIA. (**D**–**F**) The expression of NSD2 in PCa and its correlation with age, Gleason score and N stage were analysed by UALCAN.

To determine the clinical relevance of NSD2, CD4^+^ and CD8^+^ TILs, the patients with PCa were segregated into two groups according to the optimal cutoff value. As detailed in Table 3, high NSD2 expression was significantly associated with advanced Gleason score (p = 0.0001) and ISUP grade (p = 0.0001). Furthermore, high expression of NSD2 was also significantly correlated with tumor stage (p = 0.0001), lymph node metastasis (p = 0.0001) and distant metastasis (p = 0.001). Similarly, increased CD4^+^ TILs expression displayed a significant correlation with advanced Gleason score (p = 0.019) and metastasis (p = 0.016). Moreover, increased CD8^+^ TILs expression displayed a significant correlation with tumor stage (p = 0.022) and lymph node metastasis (p = 0.020) (Table 3). We evaluated the relationship between the expression of CD4 and CD8A genes and the target gene NSD2 in PCa using the GEPIA database, and the analysis showed that the expression of NSD2 was strongly correlated with the expression of CD4 (Cor = 0.26, P = 4.8e−11) and CD8A (Cor = 0.19, P = 1.7e–6), and the levels of CD4 and CD8A were closely correlated (Cor = 0.73, P = 5.6e−91) (Fig. 4A–C). Of note, in our study, significant associations were also detected between NSD2 and CD4^+^ and CD8^+^ TILs markers (p = 0.049 and p = 0.016, respectively, Table 4). Moreover, we analyzed the association of different clinical characteristics with NSD2 expression using the UALCAN database (Fig. 4D–F). In terms of age, the statistical analysis revealed significantly lower NSD2 expression in the healthy group than in patients with PCa in the 41–60 and 61–80 year age groups. NSD2 expression differed between PCa patients grouped by Gleason score and nodal metastasis status. Taken together, these data show that high expression of NSD2 might be associated with worse clinicopathological features.

**Table 3:** Correlation of NSD2 expression and CD4^+^ and CD8^+^ TILs expression with clinicopathological features.

Variables	n	NSD2			CD4^+^ TILs			CD8^+^ TILs		
		Low	High	*p* value	Low	High	*p* value	Low	High	*p* value
Age(year)	57			0.671			0.203			0.792
≤ 60	5	3	2		5	0		3	2	
> 60	52	36	16		39	13		28	24	
History of drinking	56			0.615			0.452			0.438
Yes	14	9	5		10	4		9	5	
No	42	30	12		34	8		22	20	
Gleason grade	57			0.0001*			0.019*			0.174
6–7 (3 + 4)	41	36	5		35	6		20	21	
7 (4 + 3)−10	16	3	13		9	7		11	5	
ISUP	57			0.0001*			0.204			0.098
1	9	9	0		9	0		3	6	
2	7	7	0		5	2		4	3	
3	13	12	1		9	4		4	9	
4	11	4	7		10	1		8	3	
5	17	7	10		11	6		12	5	
PSA(ng/ml)	57			0.828			0.605			0.433
≤ 20	21	14	7		17	4		10	11	
> 20	36	25	11		27	9		21	15	
Preop NLR	57			0.287			0.684			0.508
Low	46	30	16		35	11		26	20	
High	11	9	2		9	2		5	6	
Postop NLR	35			0.022*			0.139			0.486
Low	21	12	9		18	4		13	8	
High	14	13	1		9	5		7	7	
Tumor stage	57			0.0001*			0.594			0.022*
≤ T2	30	29	1		24	6		12	18	
T3–4	27	10	17		20	7		19	8	
Nodal metastasis	57			0.0001*			0.064			0.020*
Absent	15	2	13		9	6		12	3	
Present	42	37	5		35	7		19	23	
Metastasis	54			0.0001*			0.016*			0.058
Yes	19	7	12		14	9		16	7	
No	38	32	6		30	4		15	19	
Survival statue	57			0.0001*			0.005*			0.140
Dead	14	0	14		7	7		10	4	
Alive	43	39	4		37	6		21	22	
CD4^+^ TILs density	57			0.049*						0.189
Low	44	33	11					26	18	
High	13	6	7					5	8	
CD8^+^ TILs density	57			0.016*			0.189			
Low	31	17	14		26	5				
High	26	22	4		18	8				
NSD2 score	57						0.049*			0.016*
Low	39				33	6		17	22	
High	18				11	7		14	4	

Significant values are in bold.

**Table 4:** Correlation of NSD2 expression and CD4^+^ and CD8^+^ TILs marker expression in PCa tissues.

	NSD2 expression	CD4^+^ TILs density	CD8^+^ TILs density
NSD2 expression			
Spearman correlation coefficient	1.000	0.260	− 0.319
Sig		0.050	0.016*
N	57	57	57
CD4^+^ TILs density			
Spearman correlation coefficient	0.260	1.000	0.523
Sig	0.050		0.0001*
N	57	57	57
CD8^+^ TILs density			
Spearman correlation coefficient	− 0.319	0.523	1.000
Sig	0.016*	0.0001*	
N	57	57	57

Significant values are in bold.

### Overexpression of NSD2 and CD4^+^ TILs affects PCa Bone Metastasis

**Figure 5:**
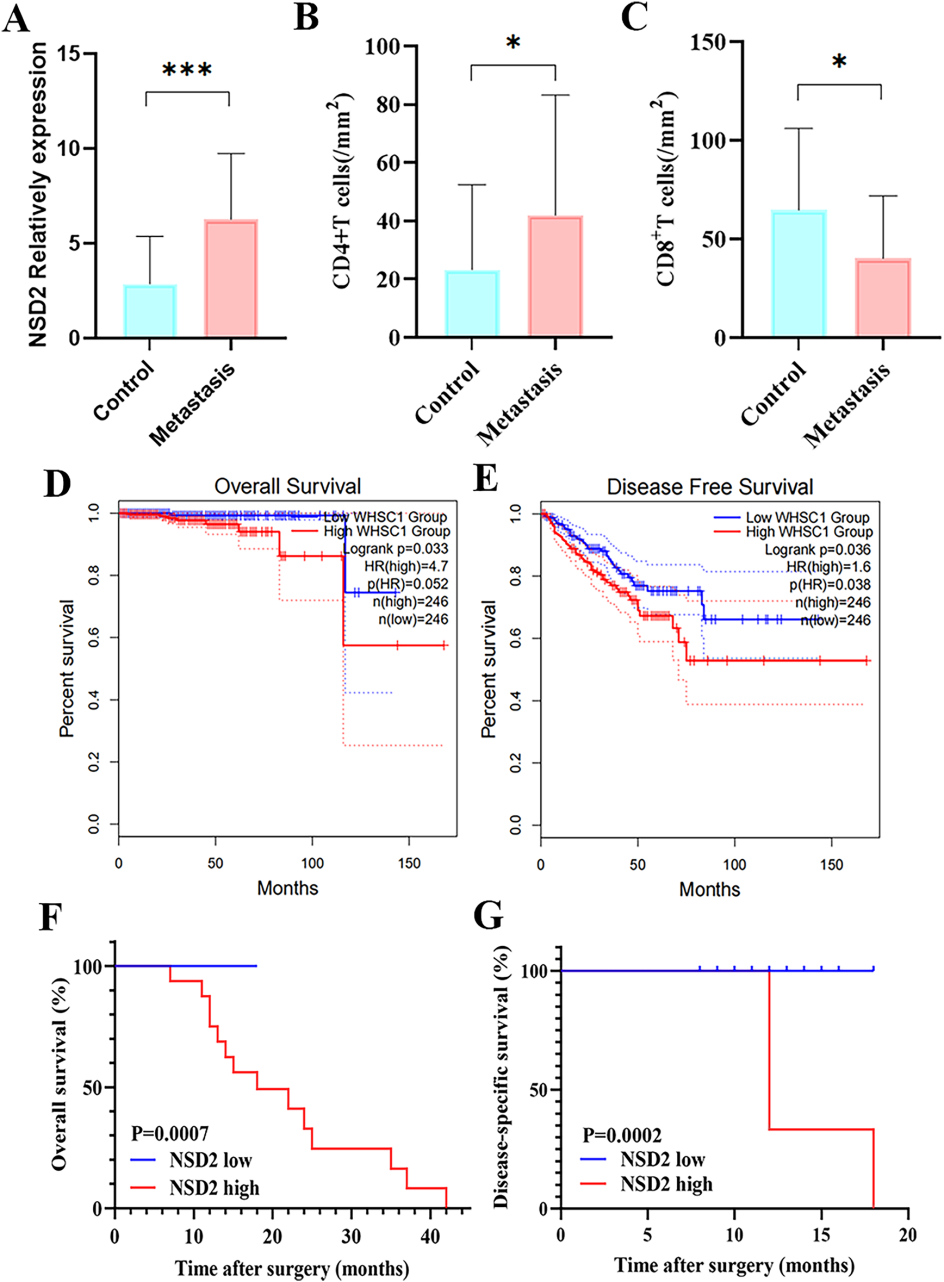
Differences in NSD2 expression, CD4^+^T, CD8^+^TILs infiltration density in the non-metastatic prostate cancer group and the prostate cancer bone metastasis group and survival analysis in PCa. (**A**–**C**) Differences in NSD2 expression, CD4^+^ and CD8^+^ TILs infiltration density in the non-metastatic prostate cancer group and prostate cancer bone metastasis group (**D**,**E**) OS and DFS of patients with PCa based on NSD2 expression determined using GEPIA. (**F**,**G**) The relationship between NSD2 expression and OS and DSS as depicted by Kaplan–Meier survival curves.

Comparison of pathological characteristics between patients with PCa bone metastasis and PCa nonbone metastasis. Bone metastasis of PCa is related to tumor stage, N stage, Gleason score, ISUP, PSA, survival status, CD4^+^ TILs density, and NSD2 expression (P < 0.05) (Table 5). Importantly, the expression of NSD2 and CD4^+^ TILs in PCa bone metastasis was significantly higher than that in PCa nonbone metastasis (NSD2, P < 0.0001; CD4^+^ TILs, P = 0.044) (Fig. 5A,B). However, a low density of CD8^+^ TILs was significantly associated with PCa bone metastasis (p = 0.0273, Fig. 5C).

**Table 5:** Basic characteristics of prostate cancer patients with and without metastasis.

Variables		Metastasis cohort	Non-metastasis	P value
		(n = 19)	(n = 38)	
		Number of cases (%)	Number of cases (%)	
Age(y, Mean range)	60(54–87)			0.331
	≤ 60	1 (5.3%)	4 (10.5%)	
	> 60	18 (94.7%)	34 (89.5%)	
History of drinking	Yes	6 (31.6%)	8 (21.6%)	0.433
	No	13 (68.4%)	29 (78.4%)	
Tumor stage	≤ T2	3 (15.8%)	27 (71.7%)	**< 0.0001**
	T3–4	16 (84.2%)	11 (28.9%)	
Nodal metastasis	Present	11 (57.9%)	4 (10.5%)	**< 0.0001**
	Absent	8 (42.1%)	34 (89.5%)	
Gleason grade	6–7(3 + 4)	1 (5.3%)	15 (39.5%)	**0.001**
	7(4 + 3)−10	18 (94.7%)	23 (37.1%)	
ISUP	1		9 (23.7%)	**0.001**
	2	1 (5.3%)	6 (15.8%)	
	3		13 (34.2%)	
	4	7 (36.8%)	4 (10.5%)	
	5	11 (57.9%)	6 (15.8%)	
PSA(ng/ml)	≤ 20	3 (10.5%)	18 (44.7%)	**0.002**
	> 20	20 (84.2%)	16 (52.6%)	
Preop NLR	Low	13 (68.4%)	29 (76.3%)	0.095
	High	6 (31.6%)	9 (23.7%)	
Survival statue	Dead	10 (52.6%)	4 (6.5%)	**0.006**
	Alive	9 (47.4%)	34 (54.8%)	
CD4^+^ TILs density	Low	12 (63.2%)	32 (84.2%)	**0.016**
	High	7 (36.8%)	6 (15.8%)	
CD8^+^ TILs density	Low	14 (73.7%)	17 (44.7%)	0.058
	High	5 (26.3%)	21 (55.3%)	
NSD2 expression	Low	7 (36.8%)	32 (84.2%)	**0.001**
	High	12 (63.2%)	6 (15.8%)	

Significant values are in bold

According to the univariate logistic regression analysis, we identified 6 prognostic factors, including PSA, Gleason score, tumor stage, nodal metastasis, CD4^+^ TIL density and NSD2 expression (P < 0.05) (Table 6). The multivariate logistic regression model analyzed these variables. The results showed that PSAand CD4^+^ TILs density (P < 0.05) were independent risk factors for bone metastasis in PCa (Table 6).

**Table 6:** Univariate and multivariate logistic analyses of prognostic factors correlated with metastasis.

Variables	Univariate analyses		Multivariate analyses	
	OR(95% CI)	P value	OR(95% CI)	P value
Age(years) ≤ 66/ > 66	2.933 0.306–28.091	0.351	1.022 0.941–1.109	0.608
PSA(ng/ml) ≤ 20/ > 20	7.500 1.872–30.046	0.004*	23.193 1.880–286.192	0.014*
NLR	0.684 0.180–2.608	0.578	0.979 0.731–1.313	0.889
Gleason score 6–7(3 + 4)/7(4 + 3)−10	8.182 2.173–30.806	0.002*	2.389 0.174–32.751	0.514
Tumor stage ≤ T2/T3–4	10.000 2.865–34.899	0.0001*	3.282 0.478–22.528	0.227
Nodal metastasis Present/Absent	11.273 2.671–47.581	0.001*	1.303 0.090–18.771	0.846
CD4^+^ TILs density Low/High	4.821 1.265–18.372	0.021*	11.983 1.158–124.054	0.037*
CD8^+^ TILs density Low/High	0.345 0.113–1.055	0.062	0.312 0.051–23.1.897	0.206
NSD2 Low/High	7.540 2.145–26.506	0.002*	2.389 0.174–32.751	0.304

Significant values are in bold.

### The prognostic significance of NSD2, CD4^+^ and CD8^+^ TILs in patients with PCa

Given the prevalence of NSD2 overexpression, the ability of NSD2 expression to predict OS was assessed by univariate analysis. Interestingly, high NSD2 expression conferred a greater than onefold increase in the risk of mortality (OS: HR = 1.267, 95% CI = 1.055–1.521, p = 0.011) (Table 7). The GEPIA database was used to investigate the NSD2 survival rate of PCa patients (Fig. 5D,E). A total of 492 patients were classified into high- or low-expression groups according to the median expression of NSD2. High NSD2 expression was associated with shorter OS (HR = 4.7, p = 0.033) and DFS (HR = 1.6, p = 0.036) in PCa patients. Further from the protein level using Kaplan–Meier analysis showed that OS and DSS was shorter in patients with high NSD2 expression than in patients with low NSD2 expression in PCa (OS: P = 0.007, DSS: P = 0.0002, respectively, Fig. 5F,G). Multivariate analysis revealed that NSD2 was not an independent risk factor for OS (Table 7). However, a high Gleason score was demonstrated to contribute to shorter survival in patients with PCa (OS: HR = 28.027, p = 0.023). By evaluating risk factors using Cox proportional hazard models, it was determined that NSD2 was a significant predictor of outcomes in PCa patients.

**Table 7:** Univariate and multivariate Cox analyses of prognostic factors correlated with OS.

Variables	Univariate analyses		Multivariate analyses	
	HR(95% CI)	P value	HR(95% CI)	P value
Age(years) ≤ 66/ > 66	1.266 0.158–10.148	0.824		
NLR	0.453 0.096–2.124	0.315		
Gleason score 6–7(3 + 4)/7(4 + 3)−10	12.107 1.471–99.608	0.020*	28.027 1.576–498.489	0.023*
Tumor stage ≤ T2/T3–4	4.958 0.599–41.066	0.138		
Nodal metastasis Present/Absent	5.542 1.092–28.118	0.039*	0.182 0.016–2.059	0.169
Metastasis Yes/No	1.682 0.408–6.929	0.472		
CD4^+^ TIL density Low/High	0.545 0.118–2.522	0.412		
CD8^+^ TIL density Low/High	0.573 0.173–1.897	0.362		
NSD2 Low/High	1.267 1.055–1.521	0.011*	1.173 0.931–1.478	0.177

Significant values are in bold.

### A novel immune classification based on NSD2, CD4^+^ and CD8^+^ TILs and its association with prognosis

**Figure 6:**
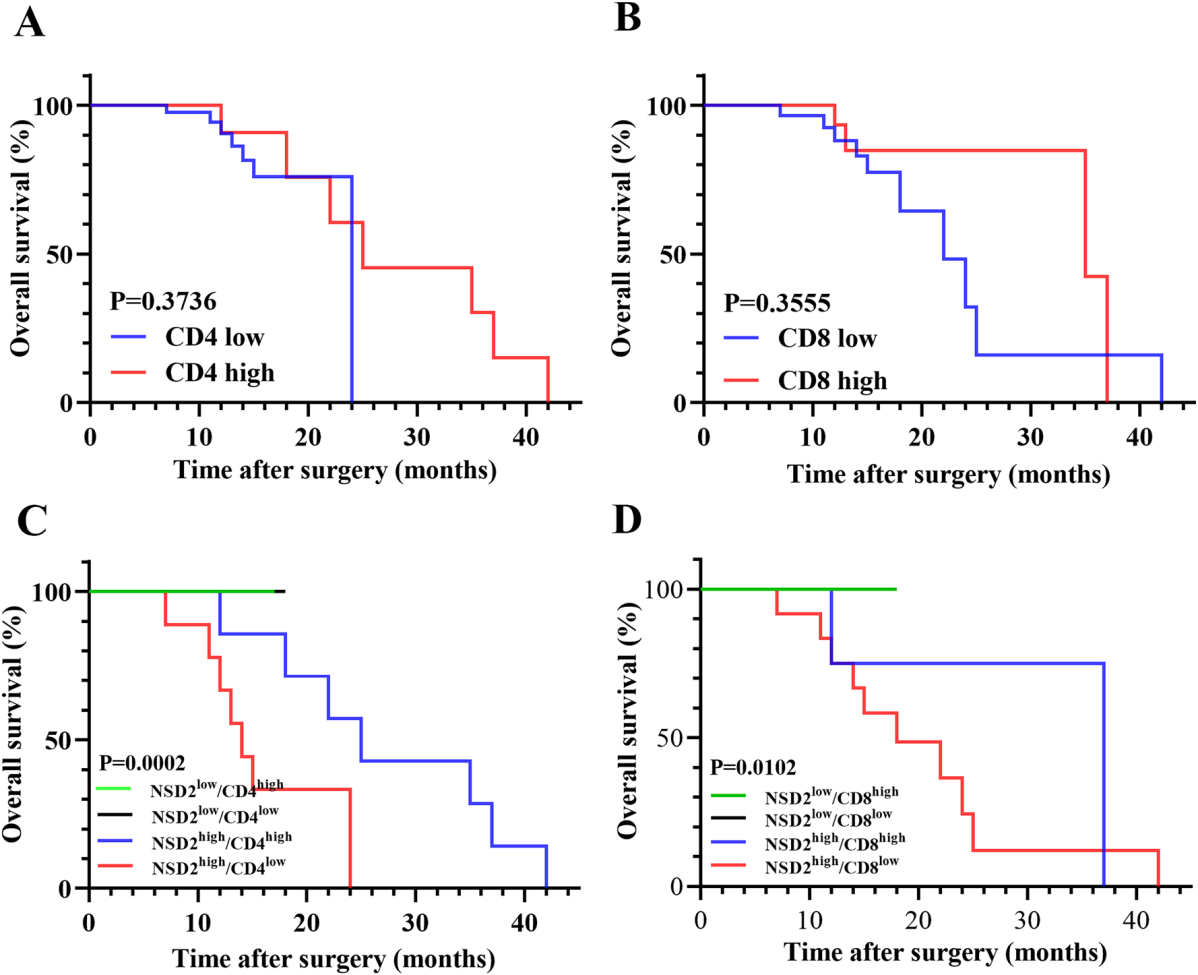
Association of new immune classification with overall survival. (**A**,**B**) OS according to CD4 and CD8 expression depicted by Kaplan‒Meier survival curves. (**C**,**D**) OS according to new immune classification in the indicated cohort.

A previous study demonstrated that extensive infiltration of CD8^+^ TILs can independently predict a favorable prognosis in PCa^25^. Our data suggest that CD4^+^ and CD8^+^ TILs did not independently predict the prognosis of PCa (Fig. 6A,B).

Considering the close correlation between NSD2 and the infiltration of CD4^+^ and CD8^+^ TILs, the combination of NSD2 expression and CD4^+^ and CD8^+^ TILs infiltration levels might better predict the prognosis of PCa. Based on the above findings, we developed a novel immune classification scheme according to NSD2 expression and the infiltration level of CD4^+^ TILs (cohort 1) and NSD2 expression and the infiltration level of CD8^+^ TILs (cohort 2). For example, the immune classification scheme for cohort 1 was immune type I (low NSD2, high CD4), immune type II (low NSD2, low CD4), immune type III (high NSD2, high CD4) and immune type IV (high NSD2, low CD4). The proportions of PCa patients with immune types I, II, III and IV were 10.5% (6/57), 57.9% (33/57), 12.3% (7/57) and 19.3% (11/57), respectively. The immunization classification scheme for cohort 2 was also consistent with the scheme for cohort 1, and the proportions of patients with PCa with immune types I, II, III and IV were 38.6% (22/57), 29.8% (17/57), 7.0% (4/57) and 24.6% (14/57), respectively. According to the Kaplan‒Meier analysis, among the four immune types, immune type IV was significantly associated with the poorest OS in both cohort 1 and cohort 2 (Fig. 6C,D). Conversely, immune types I and II were significantly correlated with improved OS in both cohort 1 and cohort 2, and no deaths occured during the follow-up. No difference in OS was found between immune type II or III in cohort 2. These data demonstrate that this novel immune classification might be useful for predicting prognosis and immunotherapy response.

### Differentially expressed gene identification and functional enrichment analysis in PCa

**Figure 7:**
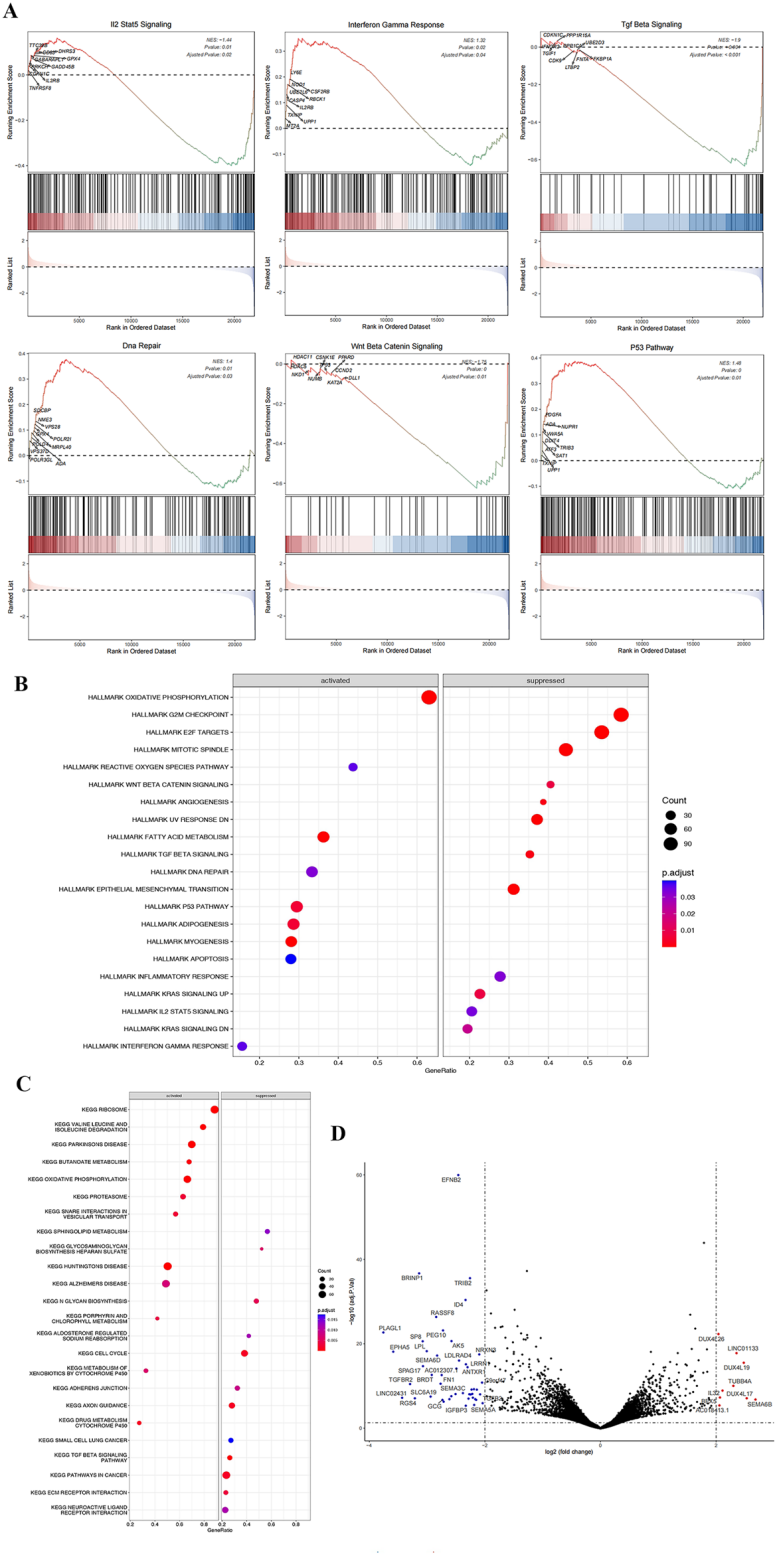
Exploratory analysis of NSD2-KO C42 cells. (**A**) Gene set enrichment analysis of HALLMARK pathways after NSD2 KO. (**B**) Gene set enrichment analysis of KEGG pathways after NSD2-KO. Circles represent the number of genes related to the term or pathway. Red and blue indicate significance; the redder the color is. (**C**) GSEA indicating pathways downregulated and upregulated following NSD2-KO. (**D**) Scatter plot of differentially expressed genes in PCa. Each dot represents a gene. Red and green dots indicate an increase or decrease, respectively, |logFC|> 0.5, and P < 0.05 in the NSD2-KO group compared to the WT group.

We then investigated which transcriptional pathways were altered in patients with high versus low NSD2 expression. GSEA was performed to identify the functional terms enriched in DEGs between the NSD2-KO and CTR groups (Fig. 7A). Single-gene GSEA revealed that NSD2-related genes are mainly enriched in the hallmark pathway and multiple pathways related to PCa development and progression, such as transition of the mitotic cell cycle pathway, Wnt/β-catenin signaling pathway, P53 signaling pathway and DNA repair pathways, corroborating the results from previous studies. The TGF-β pathway, IFN-γ response pathway, DNA repair pathway, and IL-2-STAT5 signaling pathway, which are correlated with innate and acquired immunity, were also affected by changes in NSD2 expression (Fig. 7B). The main enriched KEGG pathways were the oxidative phosphorylation pathway, cell cycle pathway, cancer pathway signaling pathway, etc. (Fig. 7C). There were 9 significantly upregulated genes and 30 significantly downregulated genes after NSD2-KO. A volcano plot was used to visualize the significantly differentially upregulated and downregulated genes in the guts of NSD2-KO mice (Fig. 7D).

### Evaluation and analysis of immune cell infiltration

**Figure 8:**
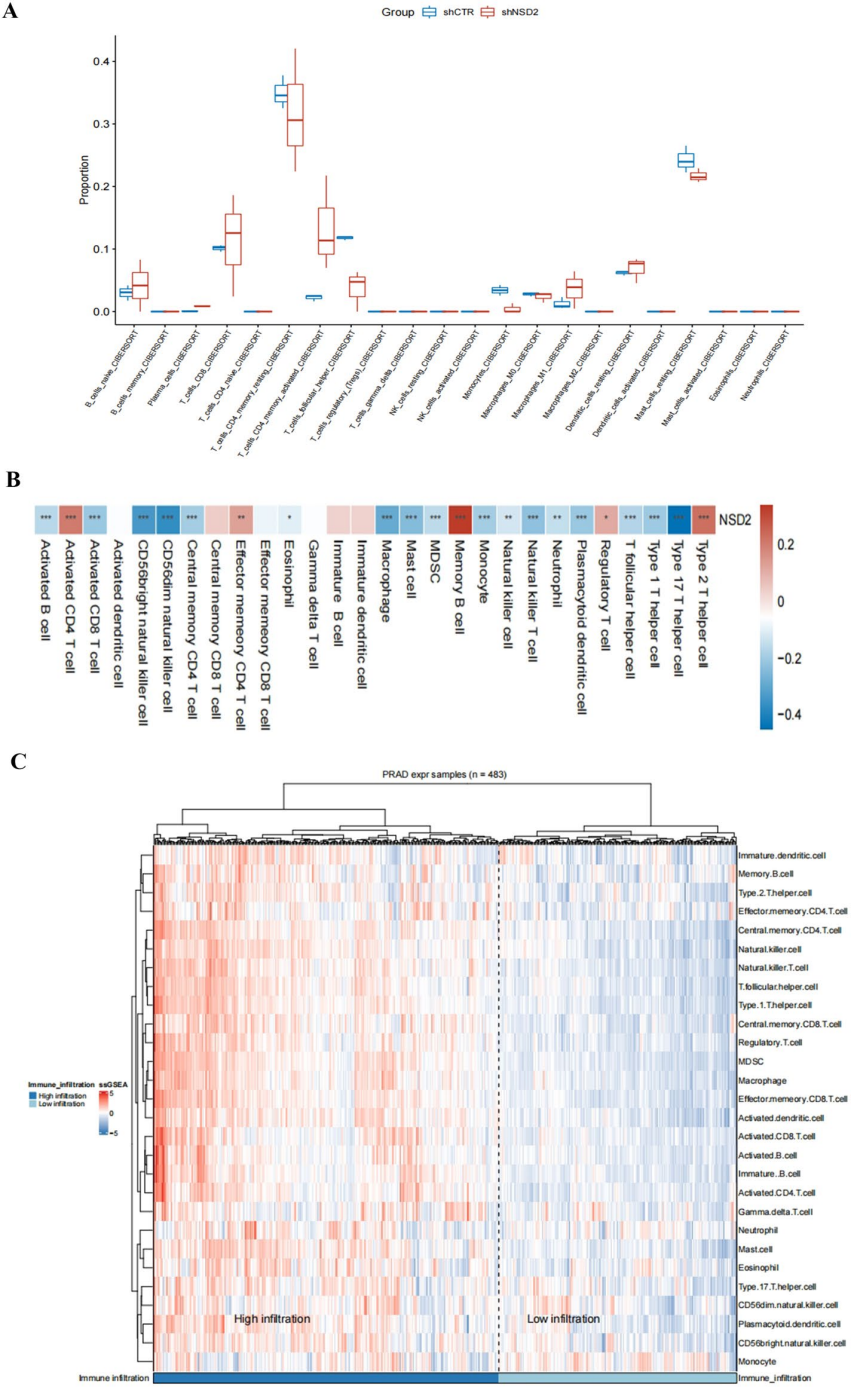
Evaluation and Analysis of Immune Cell Infiltration in PCa. (**A**) Analysis of tumor-infiltrating immune cells based on CIBERSORT algorithms. (**B**) A heatmap of NSD2 with its associated 22 immune cell types; red means positive correlation, and blue means negative correlation. A darker color indicates a stronger correlation. (**C**) Relationship between the NSD2 and immune responses in prostate cancer. Heat map of immune responses based on ssGSEA in the high infiltration group and the low infiltration group.

Next, we used six algorithms (MCPcounter, quanTiseq, xCell, CIBERSORT and EPIC) to estimate immune cell infiltration in PCa. Infiltration by follicular CD8^+^ T cells and CD4^+^ memory-activated cells, as calculated by CIBERSORT, was significantly higher in NSD2-KO samples than in NSD2-CTR samples, but unfortunately, the difference was not statistically significant (Fig. 8A). Some T-cell subtypes showed a tendency toward higher infiltration in NSD2-KO samples, as calculated by xCell, but the differences were not statistically significant. In general, the various algorithms gave consistent results for some types of T cells: their infiltration levels were higher in NSD2-KO samples.

In order to further confirm the correlation between NSD2 expression and immune cells. The CIBERSORT algorithm was used to analyze the their correlation with 28 immune cells showed that a total of 17 kinds of immune cells had significant correlations with NSD2 (Fig. 8B). Activated B cell, Activated CD8T cell, CD56bright natural killer cell, CD56dim natural killer cell, Central memory CD4 T cell, Macrophage, Mast cell, MDSC, Monocyte, Natural killer T cell, Plasmacytoid dendritic cell, T follicular helper cell, Type 1T helper cell and Type 17T helper cell were negatively correlated with NSD2 expression. There was a positive correlation between the expression of Activated CD4 T cell, Memory B cell and Type2 T helper cell and NSD2 expression. To determine whether NSD2 was related to tumor immunity, we next evaluated the association between the immune infiltration and the 28 types of TIICs in PCa from the ssGSEA. As shown in Fig. 8C, the heatmap of immune response based on ssGSEA showed that in the high infiltration group Central memory CD4T cell, Natural killer cell, Natural killer Tcell, T follicular helper cell, Type1 T helper cell, Central memory CD8T cell, Regulatory T cell, MDSC, Macrophage and Effector memory CD8T cell had higher infiltration density. Higher infiltration of CD4^+^T cell subsets than CD8^+^T cell subsets can be seen in the above highly infiltrated cells, including Central memory CD4T cell, T follicular helper cell and Type1 T helper cell. This suggests that the high infiltration group was dominated by immunosuppressive infiltrating cells, with a higher expression and density of immunosuppressive cells. These results further support that the level of NSD2 might affect the immune activity of immune cells. This result is also consistent with our experimental results that high NSD2 expression is positively correlated with high CD4^+^T infiltration.

## Discussion

PCa remains one of the deadliest cancers. Approximately 80% of patients cases with ADT therapy will progress to CRPR and eventually to mCRPR, which has a very poor prognosis, a high mortality rate, and a bone metastasis rate of 70–80%^26,27^. Currently, treatments for patients with metastatic PCa are limited. Studies have shown durable clinical responses to immunotherapeutic strategies with dendritic cell-based therapy (Sipuleucel T) and prostate-specific antigen vaccine-based immunotherapy (Prostvac), which have shown significant clinical benefit in patients with metastatic prostate cancer^28,29^. In vivo manipulations of the T-cell costimulatory pathway are also being explored as a means to evoke immune responses for the treatment of prostate cancer. However, the regulatory interface mediating T-cell infiltration into the prostate remains incompletely understood. A better understanding of PCa tumor-infiltrating lymphocytes and their microenvironment may improve our ability to target PCa immunologically, especially in view of the rapid identification of immune-related targets.

Epigenetic alterations are heritable changes that affect gene expression profiles but do not change the primary DNA sequence^30^. Investigations into aberrant epigenetic factors, including histone modifications and DNA methylation, have mainly focused on the molecular mechanisms in cancer initiation and development, new biomarkers for cancer progression and the potential of epigenetic therapy for PCa^30^. Here, we present an epigenetic tumor-driven mechanism by which prostate tumors remain relatively cold due to increased levels of the epigenetic enzyme NSD2. The role of NSD2 as a driver of metastatic prostate cancer and its involvement in AR-mediated transcription, among others, has been investigated by other groups^31,32^. Similarly, NSD2 is overexpressed in PCa, and depletion of NSD2 significantly inhibits cell proliferation, migration, and invasion, while upregulation of NSD2 can facilitate cell migration, invasion, and EMT^33^. In this study, similar observations were made. GSEA showed significant enrichment in the cell cycle pathways, such as G2M checkpoints, E2F targets and mitotic spindle, after NSD2-KO. NSD2-KO suppresses cell cycle transition and promotes apoptosis after NSD2-KO and inhibits epithelial–mesenchymal transition (EMT). Previous studies have shown that NSD2 inhibition potently enhances antigen processing and presentation via complex epigenetic remodeling of prostate cancer cells and that the IFN-γ signaling pathway increases MHC expression to stimulate antitumor immunity^14^.

To the best of our knowledge, this is the first comprehensive study comparing NSD2 expression in BPH, PIN, PCa, and mCRPC. In NSD2-stained specimens, the average staining scores were highest in Mets. Both BPH and PIN had a lower absolute staining score than PCa and Mets, although the differences were not significant. These differences support the notion that NSD2 is upregulated in many cases of prostatic adenocarcinoma. It does not appear that stronger expression occurs in all cases; 18 PCa cases had a staining score of less than 6, which was classified as low expression. In this study, through univariate analysis of relevant clinicopathological factors affecting bone metastasis in patients with PCa, it was found that PSA, Gleason score, tumor stage, nodal metastasis, CD4^+^ TILs infiltration, and NSD2 expression were related to bone metastasis (P < 0.05), while age and CD8^+^ TILs infiltration were not (P > 0.05). In addition, univariate and multivariate logistic stepwise regression analysis suggested that PSA and CD4^+^ TILs infiltration level are independent risk factors affecting the bone metastasis of PCa. Bone metastasis has been shown to be an adverse prognostic factor in many visceral malignancies^34,35^.

In addition, elevated NSD2 expression in PCa was found to correlate with virtually every adverse clinicopathologic feature and worse prognosis, as this phenotype promotes chemotherapy resistance and metastasis in PCa and other tumors, as described in previous studies and confirmed in the present study^36,37^. Specifically, high NSD2 expression was associated with higher Gleason score, ISUP grade, postoperative NLR, and pTNM stage; survival status; CD4^+^ and CD8^+^ TILs infiltration; and shorter OS. These findings provide further evidence that NSD2 might mediate immune evasion in PCa and is a potential target for immunotherapy for both primary and metastatic disease.

Accordingly, there is still an incomplete understanding of the mechanism regulating T-cell infiltration into the prostate. It is well established that TILs can have prognostic value and act as a target for cancer immunotherapy^38,39^. Although prostate cancer is a known immunogenic disease, it can escape the immune system by downregulating human leukocyte antigen class I and thereby render antigen presentation ineffective by secreting immunosuppressive cytokines such as TGF-β or by increasing regulatory T cells (Tregs)^40,41^. Similarly, an increased level of plasma TGF-β, which directly suppresses CD8^+^ T cells, was observed in bladder cancer^42^. Our data indicate that neither infiltration of CD4^+^ TILs nor infiltration of CD8^+^ TILs is associated with OS in PCa patients. A more detailed evaluation of the T-cell subpopulations in the tumor environment revealed that it is in fact the number of CD4^+^ Treg cells that predicts worse outcome in prostate cancer^43^.

In the present study, we comprehensively evaluated the association between NSD2 expression and TIL infiltration. We found that NSD2 overexpression was significantly associated with fewer CD8^+^ TILs and more CD4^+^ TILs. Furthermore, NSD2 inhibition increased the infiltration of intratumoral CD8^+^ T cells. These data demonstrate that NSD2 is associated with the immune microenvironment within PCa and support that NSD2 mainly performs immunosuppressive functions within PCa tissues. Recently, with the advent of precision medicine, immunotherapy has been proposed for a number of immune classifications. We examined the potential prognostic impact of the combined NSD2 and TIL status in patients with PCa. Our data indicate a significant association between stratification based on NSD2 expression and TIL infiltration and OS. For example, patients with a high NSD2 and less TIL infiltration may be considered to have a ‘supercold’ tumor, which is insensitive to existing clinical immunotherapy and associated with the poorest prognosis. However, it is possible that patients with low NSD2 expression and high TIL infiltration do not require subsequent adjuvant ADT and/or chemoradiotherapy because of their favorable prognosis. Combinations of therapies that can change the “cold” prostate cancer tumor microenvironment into an immunologically “hot” environment by driving T cells to the tumor may be one way to optimize immunotherapy in prostate cancer. Taken together, these results have important implications for the design of immunotherapy studies in the future.

In addition, we found that some genes in the volcano map were closely associated with the signaling pathways enriched after NSD2-KO, such as IL-32 and LINC0113, which were significantly upregulated after NSD2-KO, while IGFBP3, TGFBR2, TRIB2 and LINC02431 were significantly downregulated. IL-32 inhibits PCa growth through STAT3 and NF-κB signaling^44^. Moreover, IFN-γ can induce IL-32 production and participate in the induction of T-cell apoptosis. IGFBP3 can affect tumor cell proliferation, apoptosis, DNA damage repair and other processes through IGF-dependent or IGF-independent pathways. The lower the level of IGFBP3 in the plasma of prostate cancer patients is, the more likely they are to develop bone metastasis^45^. TGFBE2, as a member of the TGF-β-Smad signaling pathway, mediates the transduction of the TGF-β signaling pathway^46^. TRIB2 interacts with AKT, promoting its phosphorylation and blocking the p53/MDM2 pathway to promote tumor development^47^. Studies have revealed that lncRNAs play an important role in cancer signatures by participating in cell proliferation, apoptosis, migration, invasion and epithelial-mesenchymal transition (EMT)^48^. LINC01133 has been used as a developmental marker and diagnostic tool for different cancers^49^. In our study, LINC01133 was upregulated, and LINC02431 was downregulated after NSD2-KO.

Subsequently, we found in the Hallmark pathway GSEA that the DNA damage repair pathway was activated after NSD2-KO, and the IFN-γ signaling pathway promoted MHC-1 expression and antigen presentation and increased CD8^+^ T cell infiltration. Moreover, DNA damage can trigger the cGAS-STING signaling pathway to initiate type I interferon production to enhance antitumor immunity^50^. Although the TGF-β-Smad signaling pathway has tumor suppression effects, TGF-β can also accelerate tumorigenesis by enhancing immune evasion and impact multiple phases of the T-cell response^51^. For example, TGF-β signaling wis inhibited after NSD2-KO, and IFN-γ and IL-2/STAT5 signaling were activated after NSD2-KO, lead to downstream activation of acquired immunity, and therefore affects CD8^+^ T-cell proliferation^52^. Therefore, we focused on the role of NSD2 in PCa growth, migration and immune infiltration by regulating the TGF-IFN-CD4/CD8 signaling pathway. In the current study, we found that the IL-2-STAT5-5-HTP-AHR signaling pathway also contributes to CD8^+^ T-cell failure^53^. Our study provides the basis for subsequent studies on the relationship between pathways downstream of NSD2 and the TME and the mechanisms of PCa growth and metastasis.

There are several limitations involved in this study. First, as a retrospective study, selection bias is inevitable. Second, the expression levels of the immune markers evaluated in the present study were dichotomized as high or low according to the corresponding cutoff values, which were only based on the H-score in both cohorts. Thus, to increase reliability, a larger cohort is needed to identify the exact cutoff. Second, despite the significant correlations among immune markers, the exact colocalization relationships among immune markers are still unclear. Thus, multiple immunofluorescence assays are needed to confirm the associations among different immune markers. Finally, in addition to studies assessing the immunological-oncological response correlation, complementary studies by our team are necessary to further clarify the roles of other T-cell subsets, such as Tregs and T-helper CD4^+^ cells, to identify viable immune targets and related downstream pathways.

## Conclusion

In summary, we demonstrated that NSD2 is associated with an immunosuppressive microenvironment and can be an independent prognostic factor for PCa. Most importantly, we established a new immune classification system on the basis of NSD2 expression and CD4^+^ and CD8^+^ TILs marker expression, which might stratify PCa patients into group with significant differences in OS.

## Data Availability

Publicly available datasets were analyzed in this study. These data can be found here: (http://ualcan.path.uab.edu/), (http://www.proteinatlas.org/), (https://cistrome.shinyapps.io/timer/), (http://gepia.cancerpku.cn/index.html), (http://cis.hku.hk/TISIDB/index.php), (http://software.broadinstitute.org/gsea/index.jsp), (https://www.gsea-msigdb.org/gsea/msigdb), accessed on 30 April 2022 to 30 August 2022.
